# USP14 promotes the malignant progression and ibrutinib resistance of mantle cell lymphoma by stabilizing XPO1

**DOI:** 10.7150/ijms.80467

**Published:** 2023-04-01

**Authors:** Ye Zhang, Peng Lu, Shenhe Jin, Jin Zhang, Yan Zhou

**Affiliations:** 1 Department of Hematology, Sir Run Run Shaw Hospital, Zhejiang University School of Medicine, Hangzhou City, Zhejiang Province, 310016, PR. China.; 2 Department of Neurosurgery, Sir Run Run Shaw Hospital, Zhejiang University School of Medicine, Hangzhou City, Zhejiang Province, 310016, PR. China.

**Keywords:** MCL, USP14, ibrutinib resistance, ubiquitination, XPO1

## Abstract

**Background:** Mantle cell lymphoma (MCL) is a heterogeneous disease belonging to non-Hodgkin's lymphoma. In recent years, the morbidity rate of MCL is ascending, and the prognosis remains unfavorable. Ubiquitin-specific proteases14 (USP14) has been evidenced to be engaged in the process of malignant tumors. In this article, the role of USP14 in the malignant process of MCL and the mechanism of ibrutinib resistance were discussed.

**Methods:** Through qRT-PCR and western blot, the mRNA and protein expressions of USP14 in MCL cells were tested. USP14 interference plasmid was constructed by cell transfection technology, and then CCK8 and EdU assays were applied to appraise cell proliferation. Cell cycle and cell apoptosis were estimated by flow cytometry and western blot. The sensitivity of MCL cells to ibrutinib was also investigated. Next, western blot, co-IP, Cycloheximide (CHX) assay and other techniques were used to detect the relationship between USP14 and XPO1. Finally, by simultaneously inhibiting USP14 and overexpressing XPO1, the impacts of USP14 on the malignant process of MCL and the regulatory mechanism of ibrutinib sensitivity in MCL were discussed.

**Results:** USP14 expression was markedly fortified in MCL cell lines. Interference of USP14 suppressed MCL cell viability, potentiated cell cycle arrest, apoptosis, and ibrutinib sensitivity. This process might be achieved by USP14 deubiquitination through enhancing XPO1 stability.

**Conclusion:** USP14 can promote the malignant progression and ibrutinib sensitivity of MCL by stabilizing XPO1.

## Introduction

Mantle cell lymphoma (MCL) is a mature B-cell tumor that accounts for approximately 5%-10% of non-Hodgkin lymphoma. The median overall survival of MCL patients is 3-5 years, suggesting the unfavorable prognosis 1. MCL is both invasive and incurable, and it tends to occur in elderly men 2. Clinically, the main manifestation of MCL is lymphadenopathy, accompanied by hepatosplenomegaly and bone marrow infiltration 3. Since the early symptoms of MCL are not obvious and effective early diagnosis technology is lacking, patients are often diagnosed at advanced stage, making the treatment become more challenging 4. Therefore, it is urgent for researchers to explore the mechanism of MCL and find therapeutic targets for MCL.

As targeted therapy of cancer has matured, many targeted drugs have emerged, among which Ibrutinib is applied to act as a suppressor of Bruton's tyrosine kinase 5. Being the first oral targeted drug approved by Food and Drug Adminis-tration (FDA) for MCL, Ibrutinib has changed the history of hematological tumors treatment 6. However, primary drug resistance patients account for about 10.2% to 35% of all patients with ibrutinib treatment, and the proportion of acquired drug resistance patients is up to 54% 7. Resistance to ibrutinib seriously affects the efficacy of the drug. Therefore, it is of great significance to study the resistance mechanism of ibrutinib in MCL, and to find a key way to reverse the resistance and promote drug efficacy.

Ubiquitin-specific proteases14 (USP14) is a member of ubiquitin-specific proteases (USPs) family. A case of previous study has found that USP14 can dissociate ubiquitin tags from target proteins and regulate the expression levels and activities of various target proteins such as androgen receptor (AR), cell cycle-related proteins and apoptosis-related proteins in malignant tumors 8. USP14 is highly expressed in a variety of malignant tumors, and its action mechanism remains indistinct and complex, which covers the basic biological events in tumors 9-11. Meanwhile, USP14 predicts poor prognosis in most tumors 12. Therefore, USP14 can be used as a novel drug target for cancer treatment, and the development of its inhibitors is a new direction for antitumor drug research.

This article investigated the regulatory effect of USP14 on the malignant process and discussed the mechanism of ibrutinib resistance in MCL, which provided a theoretical foundation for the treatment of ibrutinib resistance in MCL.

## Materials and methods

### Cell culture

Human normal B cell line Wil2-s cells and MCL cell lines JeKo-1, Granta-519, MAVER-1 and Z138 cells that supplied from American Type Culture Collection (ATCC) were grown in RPMI1640 medium containing 10% fetal bovine serum (FBS, Invitrogen), and 1% antibiotics (Invitrogen) at 37℃ with 5% CO_2_.

### Quantitative Reverse Transcription-Polymerase Chain Reaction (qRT-PCR)

By means of Trizol reagent (Sangon Biotech), total RNA was acquired. 1 μg RNA was subjected to reverse transcription through RevertAid First Strand cDNA Synthesis Kit (Sangon Biotech), in compliance with the instructions. PCR amplification was conducted with SYBR Green Abstart PCR Mix (Sangon Biotech). Relative mRNA expression was calculated by the comparative Ct method with GAPDH serving as normalization 13.

### Western blot

RIPA buffer composed of 0.1% sodium dodecyl sulfate was applied to lyse JeKo-1 cells. Following the removal of cell debris by centrifugation at 15,000 g for 20 min at 4℃, BCA kit (Sangon Biotech) was employed to test protein concentration. PVDF membranes that were employed to move 12% SDS-PAGE-resolved equivalent amounts of protein sample were soaked in 5% non-fat milk for 1 h before cultivation with primary antibodies (1:1000, Abcam) and secondary antibody (1:5000, Abcam). Signals were finally detected using ECL (Millipore, USA) and captured employing ImageJ software.

### Cell transfection

The pcDNA3.1/USP14, USP14-specific shRNAs (shUSP14), Oe-XPO1 as well as their corresponding negative controls (Shanghai GenePharma Co.,Ltd.) were transfected into cells with Lipofectamine™ 3000 (Beijing Dingguo Changsheng Biotechnology Co., Ltd.) according to the manufacturer's instructions. After transfection for 48 h, transfection efficiency was tested with qRT-PCR and western blot. In addition, cells were treated with Ibrutinib after shRNA USP14 or over expressed the XPO1 for 48h, and the cells were grouped into control, Ibrutinib, Ibrutinib+sh-NC, Ibrutinib+sh-USP14, Ibrutinib +sh-USP14+Oe-NC, and Ibrutinib+sh-USP14+Oe-XPO1.

### Cell Counting Kit-8 (CCK8) assay

The survival of JeKo-1 cells, which were plated into 96-well plates (1×10^3^ cells per well), was determined by CCK8 (Dojindo Laboratories, Tokyo, Japan) in light of standard instructions. A microplate reader (Molecular Devices LLC, CA, USA) was applied to detect OD at 450 nm.

### 5-Ethynyl-2′-deoxyuridine (EdU) staining

Cell with indicated treatment were plated into 48-well plate (5×10^3^ cells per well) and then incubated with EdU for 2 h. Subsequently, the cells were fixed in light of the EdU kit instructions. The EdU-positive rate was recorded according to the merged pictures of EdU and DAPI.

### Flow cytometry

For cell apoptosis, in accordance with the instructions of Annexin V-PI apoptosis detection kit (Bestbio, Shanghai, China), the treated cells that suspended in 100 μl 1× Binding buffer were subjected to double staining with 5 μL Annexin V-FITC and 5 μL PI prior to the analysis with flow cytometry (BD Biosciences, USA). For cell cycle, the treated cells were immobilized in 70% ethanol prior to the incubation with RNase for 30 min at 37°C and PI (Vazyme, Nanjing, China) for 30 min at room temperature in the dark. A flow cytometer (BD Biosciences, USA) was employed to appraise cell cycle.

### Co-immunoprecipitation (Co-IP)

RIPA buffer composed of 0.1% sodium dodecyl sulfate was used to lyse JeKo-1 cells, following which 1 μg of USP14 and XPO1 antibodies (Abcam, USA) were supplemented for cultivation. 10 μL of Protein A/G Plus agarose beads were added to the lysate buffer and incubated for 2 h at 4°C with slow shaking. At last, the supernatant was removed and precooling PBS was used to wash the precipitate for several times. 2 × loading buffer was added to the precipitate and denatured for 5 min at 95°C. The supernatant was subjected to western blot to detect the target proteins.

### Cycloheximide (CHX) assay

JeKo-1 cells that seeded into 6-well plates overnight were transfected for 48 h and then exposed to 20 μM CHX. Samples at the indicated time were harvested for immunoblot analysis.

### Ubiquitination assay

JeKo-1 cells were grown in 6-well plates to achieve 80% confluence. After that, the cells were transiently co-transfected with Flag-USP14 and HA-XPO1 for 48 h with the aid of Lipofectamine™ 3000 reagent. RIPA buffer was applied to lyse the harvested cells prior to HA IP. SDS-PAGE was to resolve the input lysates and bound fractions against indicated antibodies.

### Xenograft tumor assay

The BALB/c nude mice (4-5 week, 18-20g) were injected with 2 × 10^7^ Jeko-1 cells which were transfcetd with sh-USP14 or sh-NC in the flank subcutaneously. Tumor growth was evaluated for 22 days. With the help of a caliper, tumor size was examined every 3 days according to the formula: 0.5 × (length × width). Following the sacrifice, the weight of xenograft tumors in mice was acquired. The Ethics Committee of Sir Run Run Shaw Hospital, Zhejiang University School of Medicine approved all the protocols of animal experiments in the current study.

### Immunohistochemical (IHC)

Following the deparaffinization and rehydration of paraffin-embedded tumor tissue sections (4-μm thick), 3% H_2_O_2_ was used to diminish endogenous peroxidase activity. Next, Ki-67 antibody (Abcam, USA) and biotin-labeled goat anti-mouse/rabbit IgG secondary antibodies (Abcam, USA) were incubated with the sections which were soaked in normal goat serum seal solution. Nuclear staining with DAB (Sigma Aldrich) was performed and hematoxylin was adopted for the counterstaining of the sections. IHC was performed using a kit (Beijing Zhong Shan Jin Qiao Biotechnology, Beijing, China) following the manufacturer's instructions.

### Tunel assay

With the amin of detecting the apoptosis in tumor tissues, TUNEL Assay Kit-HRP-DAB (Abcam) was used according to the manufacturer's protocols. Finally, all samples were observed under a light microscope.

### Statistical analysis

All data that analyzed with GraphPad Prism version 6.0 were presented as mean ± SD. One-way ANOVA with Tukey's post-hoc test was used in this study. P<0.05 was the threshold of significance.

## Results

### USP14 interference impeded MCL cell proliferation

**Figure 1:**
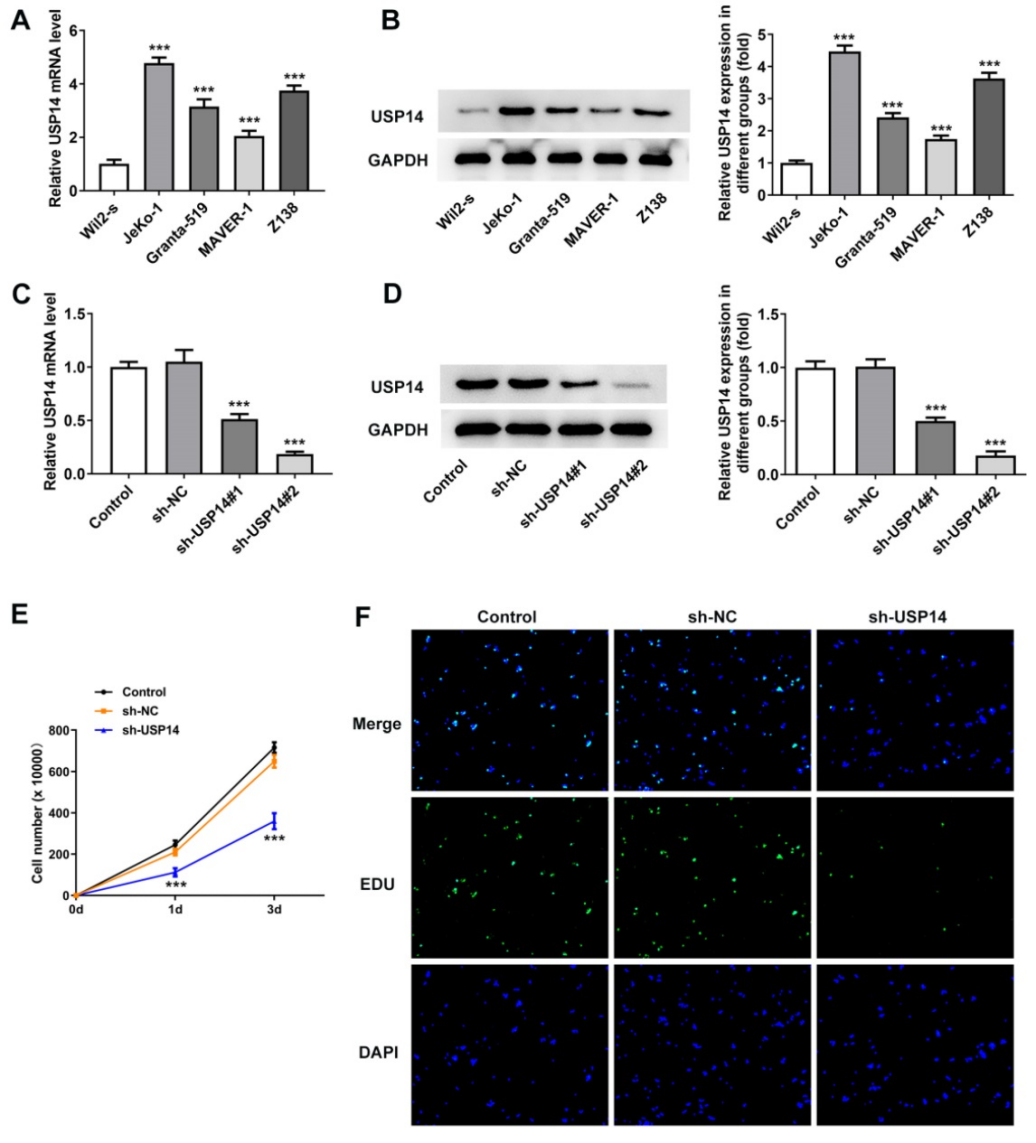
USP14 interference impeded MCL cell proliferation. qRT-PCR (A) and western blot (B) tested USP14 expression. ***P<0.001 vs Wil2-s. The transfection efficacy sh-USP14 was detected by qRT-PCR (C) and western blot (D). (E) CCK8 and (F) EdU assays judged the cell viability and proliferation respectively. ***P<0.001 vs sh-NC.

Results obtained from qRT-PCR and western blot revealed that USP14 expression was prominently augmented in MCL cell lines (Fig. 1A and B). It was noted that USP14 had the highest expression in JeKo-1 cells compared with other MCL cell lines, in this way, JeKo-1 cells were selected for subsequrnt experiments. USP13 expression was tested via qRT-PCR and western blot after the transfection with USP14 interference plasmids (Fig. 1C and D). CCK8 and EdU staining results corroborated that relative to sh-NC group, the viability and proliferation of JeKo-1 cells were markedly diminished in the sh-USP14 group (Fig. 1E and F).

### Interference with USP14 induced G2/M cycle arrest and apoptosis of MCL cells

**Figure 2:**
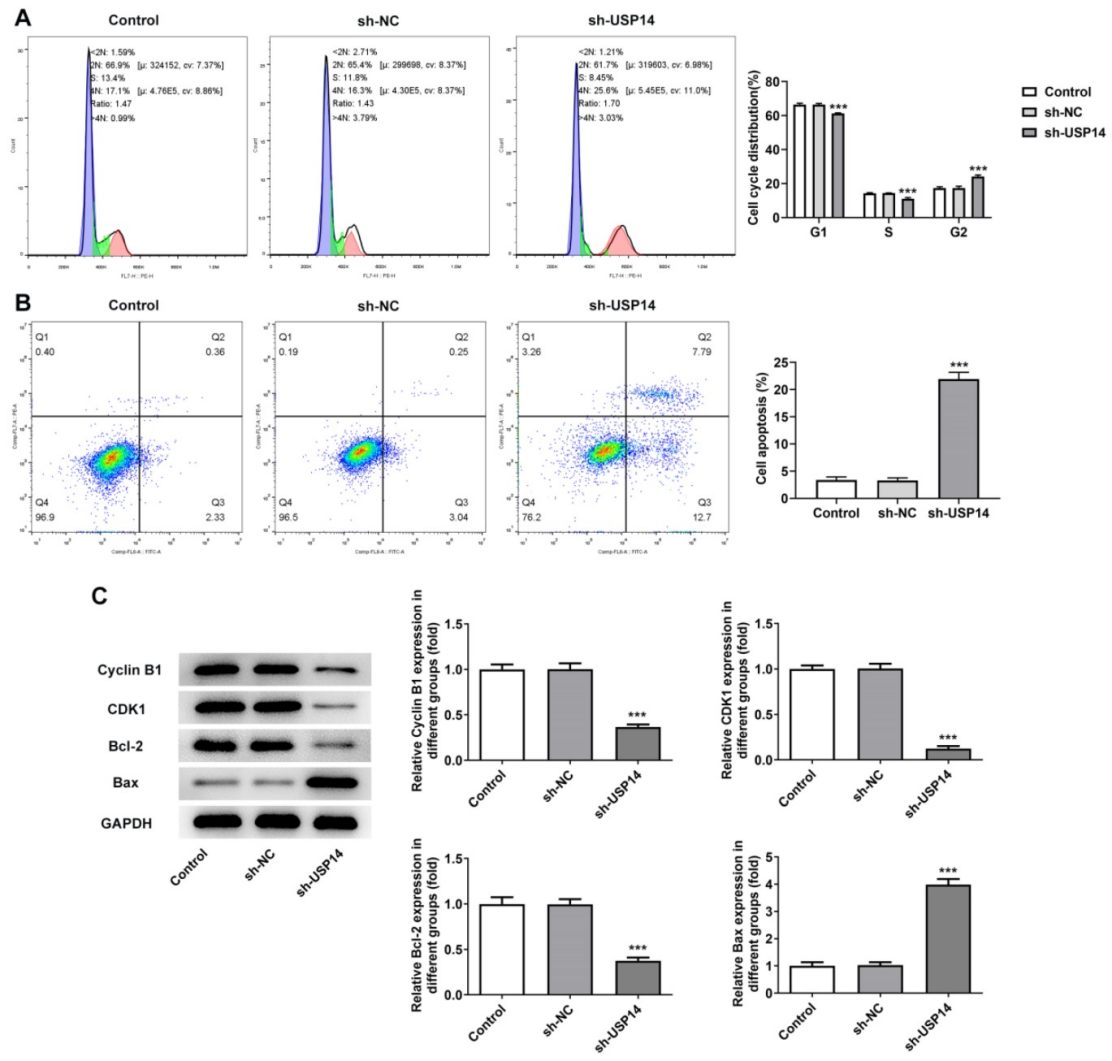
Interference with USP14 induced G2/M cycle arrest and apoptosis of MCL cells. Flow cytometry analysis measured cell cycle (A) as well as apoptosis (B). Analysis of the proteins associated with cycle and apoptosis by western blot. ***P<0.001 vs sh-NC.

Flow cytometry results presented that relative to the sh-NC group, the cell cycle arrest and apoptosis were notably exacerbated in the sh-USP14 group (Fig. 2A and B). Western blot results manifested that the expressions of cell cycle-associated proteins Cyclin B1 and CDK1 were distinctly declined, the expression of anti-apoptotic protein Bcl-2 was reduced, while the expression of pro-apoptotic protein Bax was conspicuously raised after USP14 was silenced (Fig. 2C).

### Interference with USP14 increased the sensitivity of MCL cells to ibrutinib

**Figure 3:**
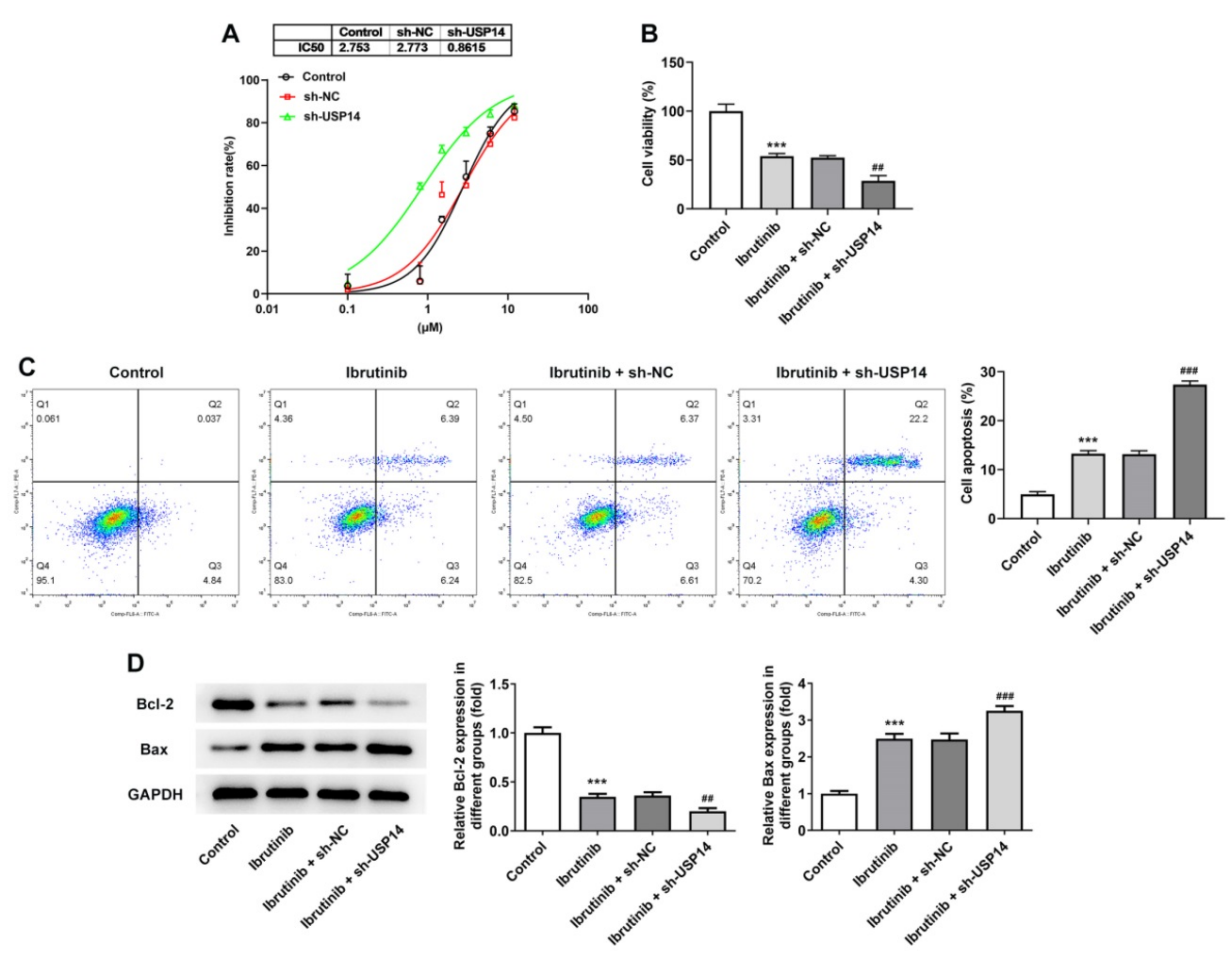
Interference with USP14 increased the sensitivity of MCL cells to ibrutinib. A. CCK8 estimated the cell inhibition rate. B. CCK8 judged cell viability. C. Flow cytometry measured cell apoptosis. D. Western blot tested Bcl-2 and Bax expressions. ***P<0.001 vs Control; ##P<0.01, ###P<0.001 vs Ibrutinib + sh-NC.

As CCK8 depicted, the level of IC50 in the sh-USP14 group was apparently reduced by contrast with that in the sh-NC group (Fig. 3A). Moreover, the cell viability was notably suppressed after the administration of ibrutinib. Relative to the Ibrutinib+sh-NC group, the cell viability in the Ibrutinib+sh-USP14 group was further diminished (Fig. 3B). Flow cytometry and western blot results illuminated that after the treatment with ibrutinib, the apoptotic rate was remarkably elevated, accompanied by the descending Bcl-2 expression and the ascending Bax expression. Relative to Ibrutinib+sh-NC group, apoptosis was obviously potentiated in Ibrutinib+sh-USP14 group, accompanied by further descending Bcl-2 expression and ascending Bax expression (Fig. 3C and D).

### USP14 stabilized XPO1 through deubiquitination of XPO1

**Figure 4:**
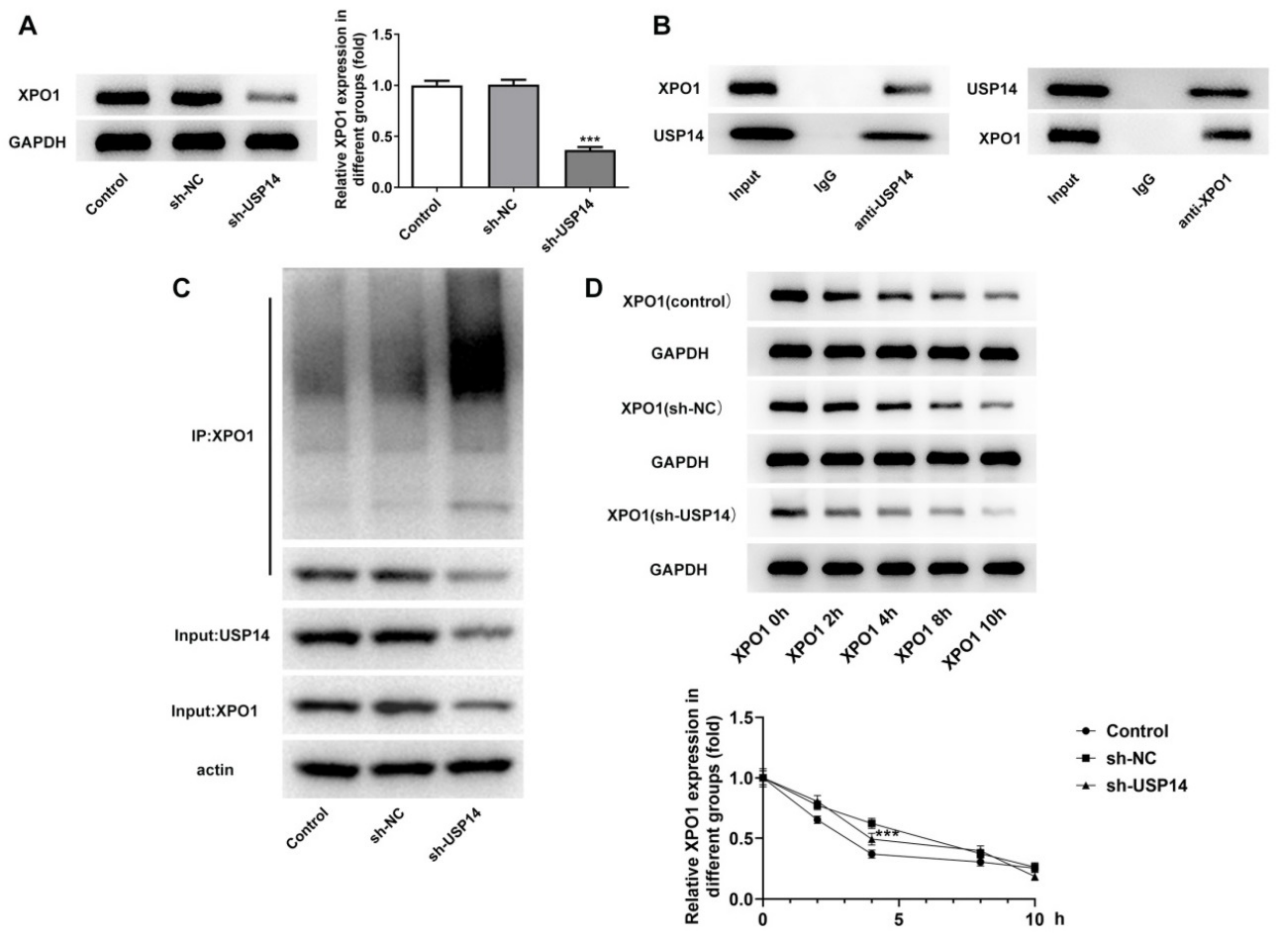
USP14 stabilized XPO1 through deubiquitination of XPO1. A. Western blot detected the expression of XPO1 after the inhibition of USP14. B. Co-IP proved that USP14 could interact with XPO1. C. CHX assay detected ubiquitination. D. USP14 knockdown increased the ubiquitination of XPO1 in MCL cells. ***P<0.001 vs sh-NC.

Western blot analysis uncovered that XPO1 expression was prominently cut down after depleting USP14 expression relative to the sh-NC group (Fig. 4A). Co-IP results showed that USP14 could interact with XPO1 (Fig. 4B). In addition, the inhibition of USP14 increased ubiquitination of XPO1 protein (Fig. 4C). The half-life of endogenous XPO1 protein was markedly shortened on account of USP14 knockdown (Fig. 4D), indicating that XPO1 protein degradation was stimulated by USP14 deficiency.

### Overexpression of XPO1 reversed the effect of USP14 downregulation on MCL cells

**Figure 5:**
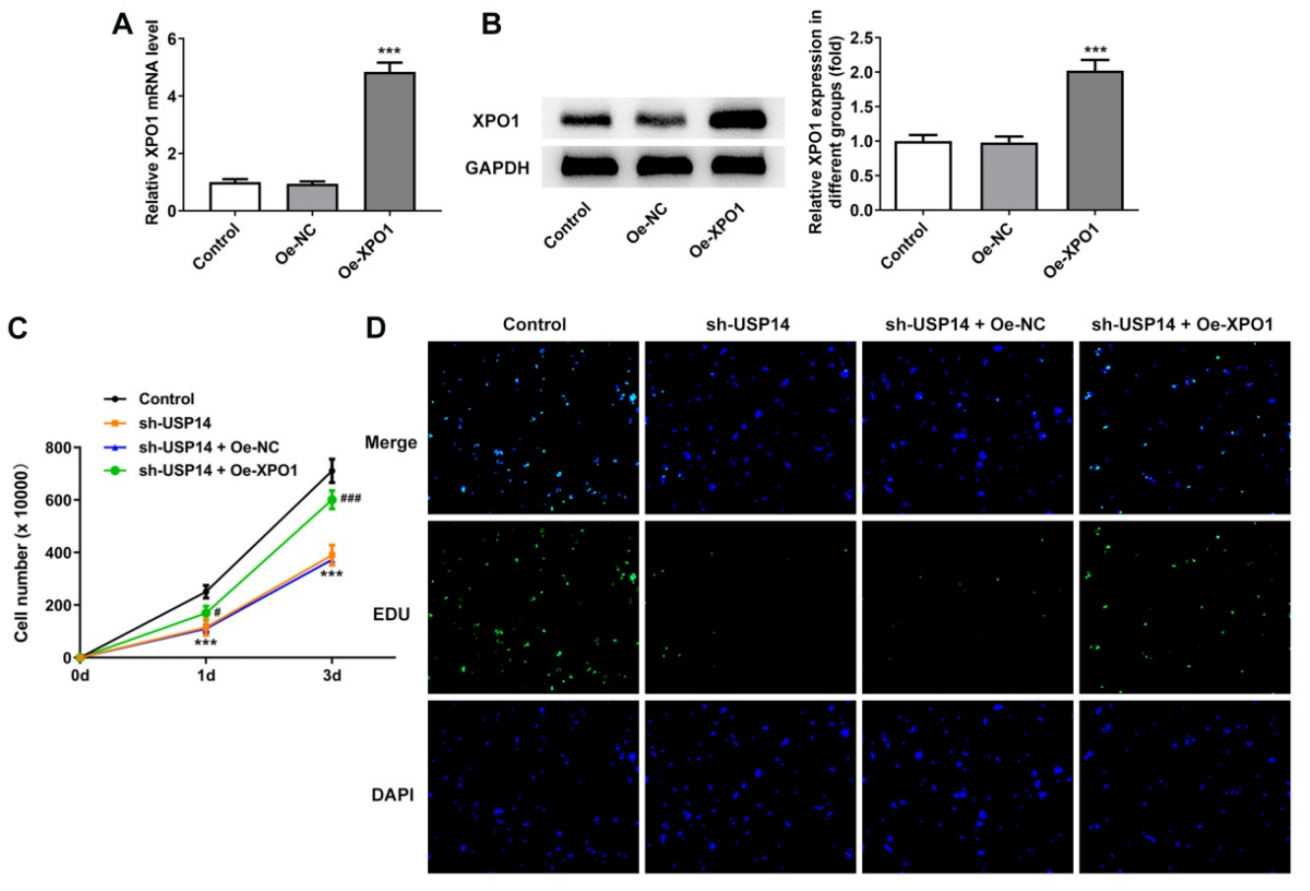
XPO1 elevation reversed the impact of USP14 deficiency on MCL cell proliferation. qRT-PCR (A) and western blot (B) tested XPO1 expression. ***P<0.001 vs Oe-NC. (C) CCK8 and (D) EdU assays judged the cell viability and proliferation respectively. ***P<0.001 vs Control; ###P<0.001 vs sh-USP14 + Oe-NC.

**Figure 6:**
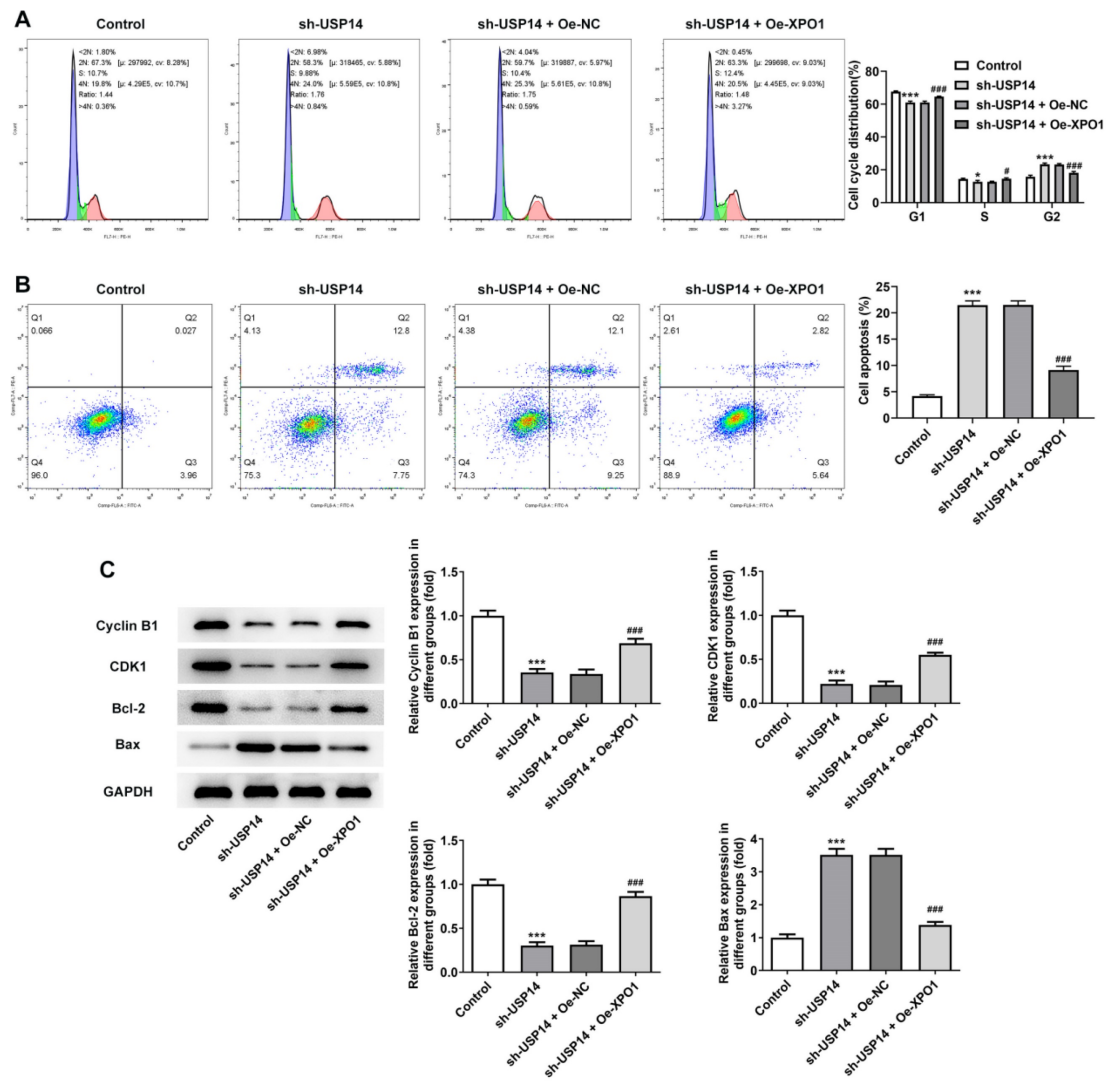
XPO1 elevation countervailed the impact of USP14 silencing on MCL cell cycle and apoptosis. Flow cytometry analysis measured cell cycle (A) as well as apoptosis (B). C. Analysis of the expression of proteins associated with cycle and apoptosis by western blot. *P<0.05, ***P<0.001 vs Control; #P<0.05, ###P<0.001 vs sh-USP14 + Oe-NC.

XPO1 expression was noted to be greatly elevated following the construction of XPO1 overexpression plasmid (Fig. 5A and B). CCK8 and EdU results corroborated that relative to the sh-USP14+Oe-NC group, the cell viability and proliferation of the sh-USP14+Oe-XPO1 group were prominently facilitated (Fig. 5C and D). Flow cytometry results revealed that by contrast with the sh-USP14+Oe-NC group, the cell cycle arrest was obstructed and the apoptosis was noticeably hindered in the sh-USP14 + Oe-XPO1 group (Fig. 6A and B). Also, relative to the sh-USP14+Oe-NC group, Cyclin B1, CDK1 and Bcl-2 expressions were notably fortified, while Bax expression was evidently lessened in the sh-USP14 + Oe-XPO1 group (Fig. 6C).

**Figure 7:**
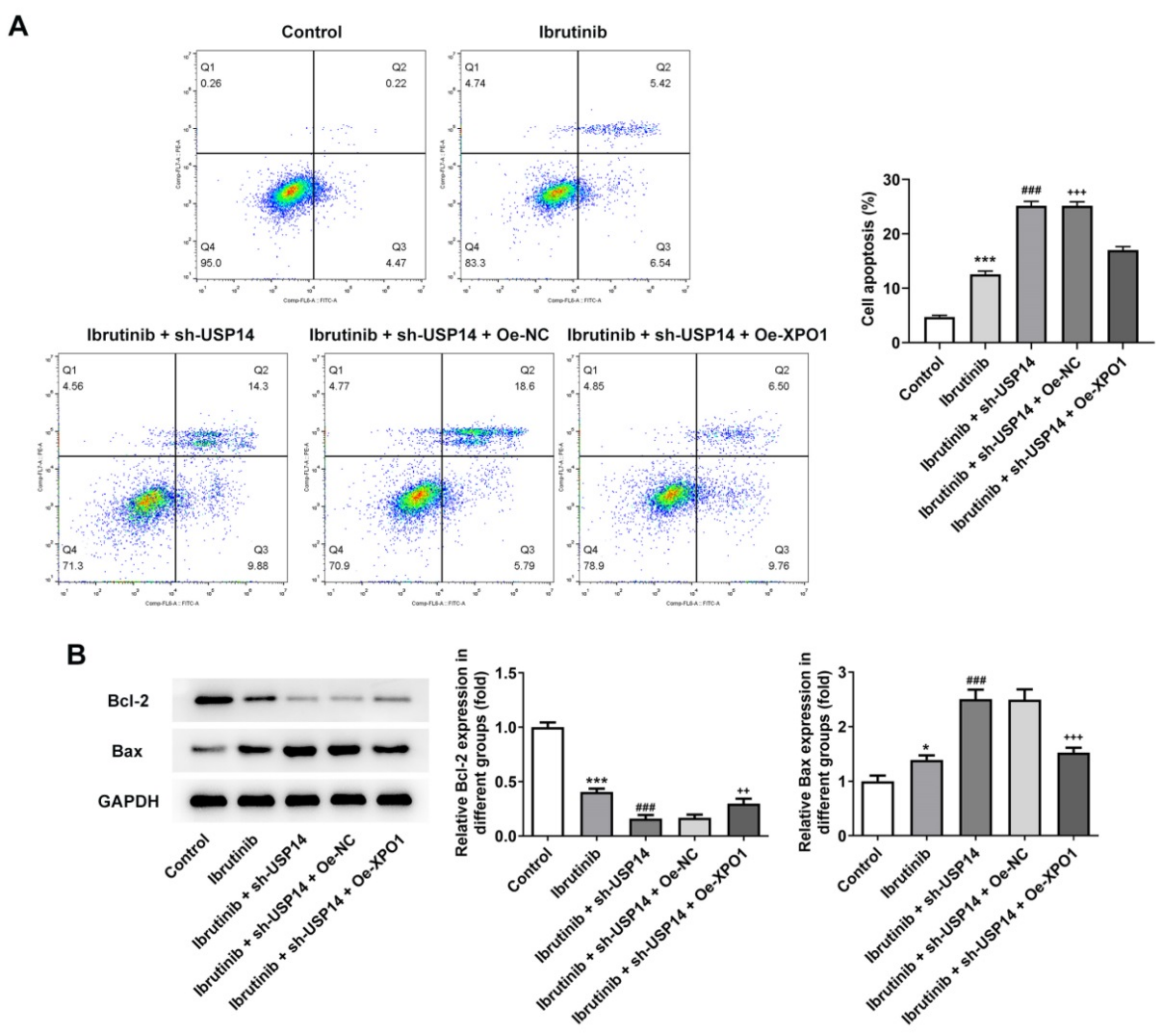
Overexpression of XPO1 reversed the effect of USP14 downregulation on the sensitivity of MCL cells to ibrutinib. A. Flow cytometry measured cell apoptosis. B. Western blot tested Bcl-2 and Bax expressions. *P<0.05, ***P<0.001 vs Control; ###P<0.001 vs Ibrutinib; ++P<0.01, +++P<0.001 vs Ibrutinib + sh-USP14 + Oe-NC.

Subsequently, the regulatory mechanism of USP14 on the sensitivity of MCL cells to ibrutinib was investigated. Flow cytometry results elucidated that relative to Ibrutinib+sh-USP14+Oe-NC group, the apoptosis rate of Ibrutinib+sh-USP14+Oe-XPO1 group was overtly declined (Fig. 7A). By contrast with Ibrutinib+sh-USP14+Oe-NC group, Bcl-2 expression was enhanced and Bax expression was lessened in Ibrutinib+sh-USP14+Oe-XPO1 group (Fig. 7B).

### USP14 down-regulation inhibited MCL cell growth *in vivo*

**Figure 8:**
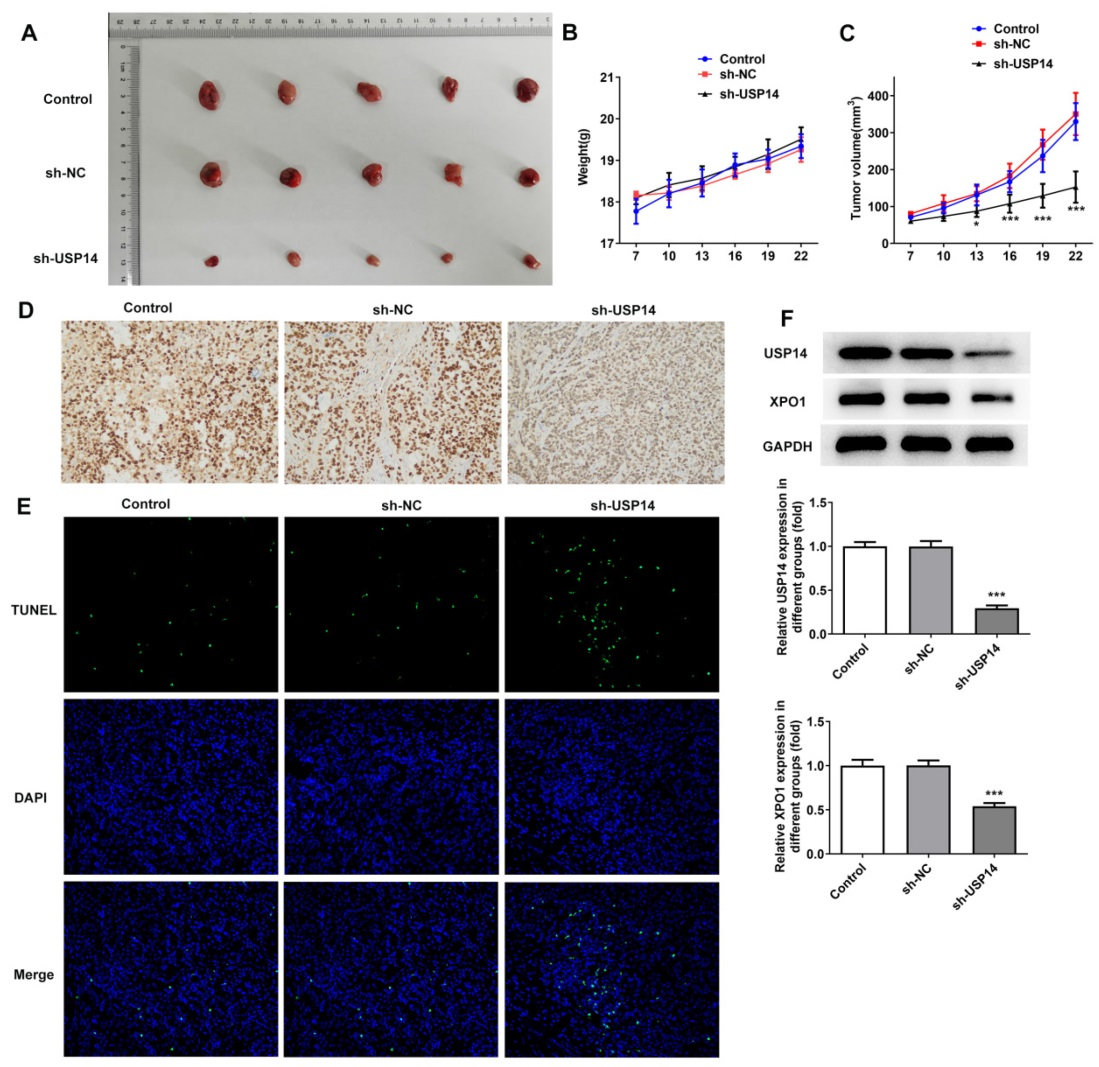
USP14 down-regulation inhibited MCL cell growth *in vivo*. A. The images and weights of mice in each group. B and C. Image of tumor volume and size. D. IHC assay examined Ki-67 expression. E. Tunel assay detected the apoptosis rate of tumor cells *in vivo*. F. Western blot detected the expressions of USP14 and XPO1 in the tumor tissues. ***P<0.001 vs sh-NC.

Then, we investigated the regulatory role of USP14 in MCL *in vivo*. The photos of tumor tissues were shown in Figure 8A. USP14 absence prominently suppressed tumor growth in MCL mice by contrast with the sh-NC group (Fig. 8B and C). IHC results implied that deficiency of USP14 declined Ki-67 expression in tumor tissues (Fig. 8D). Tunel results uncovered that relative to sh-NC group, the apoptotic capacity in sh-USP14 group was obviously elevated (Fig. 8E). Additionally, relative to the sh-NC group, USP14 and XPO1 expressions in the tumor tissues were declined in the sh-USP14 group (Fig. 8F).

## Discussion

As one of the main members of ubiquitin proteasome system, USP14 has attracted much attention because of its important role in a variety of cancers. A previous study has highlighted that USP14 contributed to cell growth, migration, the activation of kinase and inflammasome, as well as inhibited autophagy and apoptosis in cancer cells 14. USP14 had elevated expression in epithelial ovarian cancer tissues, and the knockdown of USP14 could induce cell apoptosis through the Bcl-xl pathway to inhibit cell proliferation 12, 15. In addition, a study has shown that USP14 could down-regulate cyclins and activate aspartic proteases-dependent apoptotic pathways, thereby blocking the growth of myeloma cells 16. Huang G et al. confirmed that USP14 expression was markedly augmented in liver cancer cells and USP14 absence could hamper cell proliferation, drive apoptosis and result in the altered cell cycle of liver cancer cells 17. Similarly, high expression of USP14 was significantly associated with distal metastasis in esophageal squamous cell carcinoma 18. To conclude, USP14 expression was elevated in various cancers 19. In our experimental results, USP14 expression in MCL cell lines JeKo-1, Granta-519, MAVER-1 and Z138 cells was abnormally fortified, which was also in agreement with the results of other studies. Subsequently, USP14 expression was silenced in MCL cells and it was found that cell viability was decreased, cell cycle arrest as well as apoptosis was potentiated.

Ibrutinib has been extensively implicated in the therapy for MCL, but its resistance is also extremely restrictive for the treatment of MCL 20. At present, there are few discussions regarding on the mechanism of ibrutinib resistance in MCL. Through literature review, it was found that USP14 played an important role in the mechanism of chemotherapeutic drug resistance. The study showed that USP14 inhibitor could inhibit tumor growth in melanoma *in vivo* and increase the sensitivity of melanoma to vemulafenib 21. Inhibition of USP14 enhanced breast cancer sensitivity to enzalutamide 22. USP14 was involved in cell adhesion-mediated drug resistance in multiple myeloma by acting as a bridge between Bcl-xl apoptosis pathway and Wnt signaling pathway 23. Inhibition of USP14 promoted connexin 32 internalization and counteracted cisplatin cytotoxicity in human ovarian cancer cells 24. In addition, US14/UCHL5 inhibitors could cause specific apoptosis in bortezomib or ibrutinib-resistant Waldenstrom macroglobulinemia cells, suggesting that USP14 could be used as a new therapeutic target for Waldenstrom macroglobulinemia 25. In our experiments, it was found that the sensitivity of MCL cells to ibrutinib was significantly increased after depleting USP14 expression, implying that USP14 participated in ibrutinib resistance in MCL.

BioGRID database predicted a potential interaction between USP14 and XPO1. Studies have shown that XPO1 played a cancer-promoting role in various cancers 26-28. Moreover, XPO1 inhibitor Selinfor overcomeed ibrutinib resistance in mantle cell lymphoma through nuclear retention of IκB 29. Elevated XPO1 expression predicted poor prognosis of MCL patients. XPO1 inhibitor PKT-185 induced MCL cell apoptosis through p53-dependent and independent mechanisms 30. In addition, USP14 promoted prostate cancer progression through deubiquitinating the transcriptional factor ATF2 31. Therefore, we hypothesized that USP14 played a role in the malignant process of MCL and ibrutinib resistance by binding to XPO1. First, we confirmed that USP14 enhanced XPO1 stability through deubiquitination via Co-IP and ubiquitination-related experiments. Subsequently, we investigated the mechanism of USP14 and XPO1 in the malignant progression of MCL and ibrutinib resistance by cell transfection technology. We found that overexpression of XPO1 reversed the effects of USP14 down-regulation on MCL cell proliferation, cell cycle arrest, apoptosis as well as ibrutinib sensitivity. The regulatory role of USP14 in MCL was further verified in animal experiments.

## Conclusion

USP14 contributed to the malignant progression of MCL and ibrutinib resistance by stabilizing XPO1.
